# Endoscopic Ultrasound-Guided Tissue Sampling for the Cytohistological Diagnosis of Focal Liver Lesions

**DOI:** 10.3390/diagnostics14111155

**Published:** 2024-05-31

**Authors:** Jose Lariño-Noia, Andrea Jardi-Cuadrado, Juan Enrique Dominguez-Muñoz, Yessica Domínguez-Novoa, Marco Galego, Alberto Rama, Daniel de la Iglesia-Garcia, Xurxo Martinez-Seara, Ihab Abdulkader-Nallib, Julio Iglesias-Garcia

**Affiliations:** 1 Department of Gastroenterology and Hepatology, Health Research Institute of Santiago de Compostela (IDIS), University Hospital of Santiago de Compostela, 15706 Santiago de Compostela, Spain; andreajardi@hotmail.com (A.J.-C.); enriquedominguezmunoz@hotmail.com (J.E.D.-M.); yessicadomingueznovoa@gmail.com (Y.D.-N.); marcogalegofdez@gmail.com (M.G.); albertorama95@gmail.com (A.R.); danieldelaiglesiagarcia@gmail.com (D.d.l.I.-G.); xurxoensayos@gmail.com (X.M.-S.); julioiglesias.garcia@usc.es (J.I.-G.); 2 Department of Pathology, Health Research Institute of Santiago de Compostela (IDIS), University Hospital of Santiago de Compostela, 15706 Santiago de Compostela, Spain; ihab.abdulkader.nallib@sergas.es

**Keywords:** endoscopic ultrasound, focal liver lesions, diagnostic yield

## Abstract

Background: Focal liver lesions (FLL) often require cytohistological evaluation. Endoscopic Ultrasound (EUS)-guided tissue acquisition (EUS-TA) is highly accurate in diagnosing pancreatic and gastrointestinal malignancies. The aim of our study was to evaluate the role of EUS-TA in the characterization of FLL. Methods: A retrospective analysis of a prospective database of patients who underwent EUS-TA for the evaluation of FLL. Diagnostic yield, adverse events and factors associated with diagnostic yield were evaluated as endpoints. The effect of variables such as needle size, lesion size, rapid on-site evaluation (ROSE) and the use of cytological or histological needles were analyzed. Results: A total of 114 cases were included (mean age 68.05 ± 11.35 years, 64 male). A correct diagnosis was made using EUS-TA in 100 of the 114 cases (diagnostic yield of 88%). The EUS-TA of additional extrahepatic lesions during the same EUS procedure increased the diagnostic yield to 94%. No adverse events were reported. Multivariate analysis did not identify any factor influencing the diagnostic yield. Conclusions: EUS-TA is a highly accurate and safe technique for the differential diagnosis of FLL and could be considered as the primary approach in this setting.

## 1. Introduction

Percutaneous abdominal ultrasound (US), multidetector computed tomography or magnetic resonance imaging have been used over the years for the diagnosis and evaluation of focal liver lesions and diffuse liver diseases. Although these imaging techniques have presented and developed multiple advances in recent years, cytohistological evaluation is often required to characterize focal liver lesions. This is mainly related to lesions with atypical imaging findings, certain tumors that are difficult to differentiate between benign and malignant, or metastatic liver lesions of various malignant neoplasms that typically require cytohistological evaluation.

US-guided biopsy is nowadays considered the standard method and is the most performed procedure in clinical practice [1,2]. However, US-guided biopsy has certain limitations as it cannot target all locations (i.e., lesions located in the deep portion of segment VIII) and can sometimes miss lesions smaller than 1–2 cm [3].

Endoscopic ultrasound (EUS) has become a popular technique for the evaluation of liver disease in recent years [4,5,6]. The proximity of the liver to the gastrointestinal lumen allows accurate and safe targeting and the evaluation of both lobes of the liver. Advanced imaging techniques associated with EUS, such as EUS-guided elastography and/or contrast-enhanced harmonic EUS, have also been shown to be useful for the better characterization of liver diseases, including focal liver lesions. However, the key advantage of EUS is the possibility of performing guided tissue acquisition. EUS-guided fine needle aspiration (FNA) and fine needle biopsy (FNB) are commonly used to target solid and cystic lesions in the liver, which can be accessed via a transgastric or transduodenal approach [7]. Recently, EUS has been shown to provide adequate liver samples for the histological evaluation of parenchymal liver disease, according to current guidelines [8] and the assessment of the portal pressure gradient during the same procedure [9].

Although EUS-guided tissue acquisition has been shown to have a good diagnostic yield and safety profile for the evaluation of focal liver lesions, it is still considered a rescue technique in clinical guidelines when percutaneous US is not possible (either for difficult access for the specific liver lesions or related to different technical issues) or has failed [10]. However, reported studies have included small numbers of patients and do not reflect recent improvements in EUS-guided tissue acquisition. 

The aim of our study was to evaluate the diagnostic yield and safety of the EUS-guided tissue acquisition of focal liver lesions in a large cohort of patients.

## 2. Materials and Methods

This is a retrospective cohort study of patients prospectively enrolled in an EUS database at the Endoscopy Unit of the Department of Gastroenterology and Hepatology, University Hospital of Santiago de Compostela, Spain. The analysis included patients diagnosed with focal liver lesions, who underwent EUS-guided tissue acquisition between January 2013 and December 2023. The study design, data collection, and analysis adhered to the tenets of the STROBE statement [11].

### 2.1. Patients and EUS-Guided Tissue Acquisition Method

All patients with a focal liver lesion who underwent EUS-guided tissue acquisition during the study period were initially included. Focal liver lesions were identified either before EUS using percutaneous abdominal ultrasound or abdominal computed tomography or during the same EUS procedure.

At our center, the general policy is to perform percutaneous US-guided biopsy for those focal liver lesions located in the right hepatic lobe and EUS-guided tissue acquisition for those lesions located in the left hepatic lobe. In addition, EUS-guided tissue acquisition is performed for any liver lesion that is primarily detected on EUS, regardless of its location.

**Figure 1:**
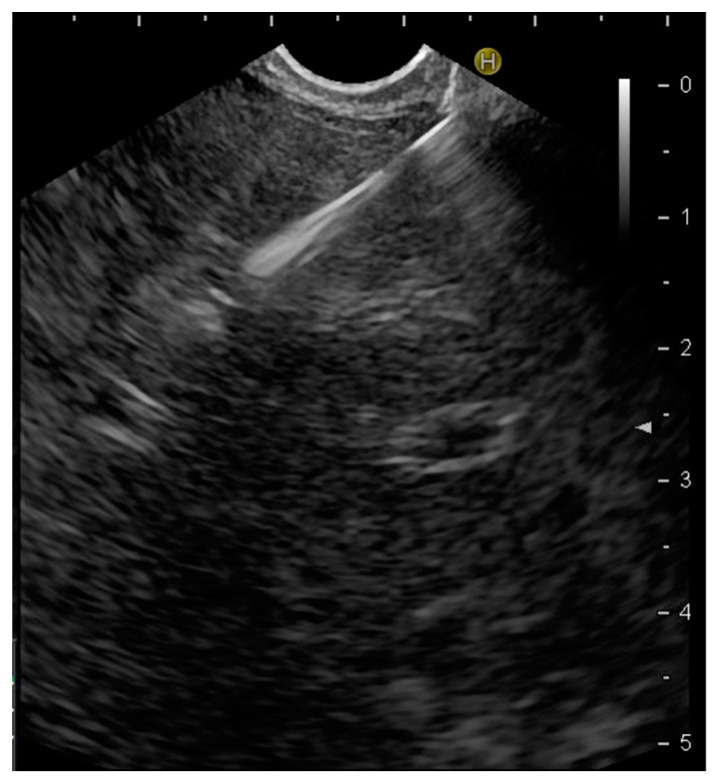
EUS-guided tissue sampling from a solid liver lesion, which corresponds to a liver metastasis from colorectal cancer.

EUS was performed in the left lateral decubitus position under conscious or deep sedation using a linear array echoendoscope (Pentax 38J10, Pentax Europe GmbH, Hamburg, Germany EG) attached to an ultrasound machine (Hitachi Medical Systems Europe, Zug, Switzerland). Cytological or core needles ranging from 19 to 25 gauge (G) with different tip designs (Franseen, reverse-bevel or fork tip) were used for EUS-guided tissue acquisition, depending on availability and endosonographer preference. After a careful EUS evaluation of the target lesion and regional vasculature, including real-time Doppler, tissue acquisition was performed either from the stomach to the left liver lobe or from the duodenum to the right hepatic lobe. The needle was advanced into the target lesion under EUS guidance (Figure 1). Once the lesion was penetrated, the stylet was removed. The sample was obtained by moving the needle back and forth within the lesion while applying negative pressure using a 10 mL syringe. The suction was released by closing the syringe lock and the needle was finally removed. If a sample obtained by the first needle pass was bloody, a second needle pass was performed without applying negative pressure. Patients were observed in the recovery room for about 2 h for immediate complications and followed for 6 months for potential late adverse events. No prophylactic antibiotics were given.

### 2.2. Cytohistological Evaluation

**Figure 2:**
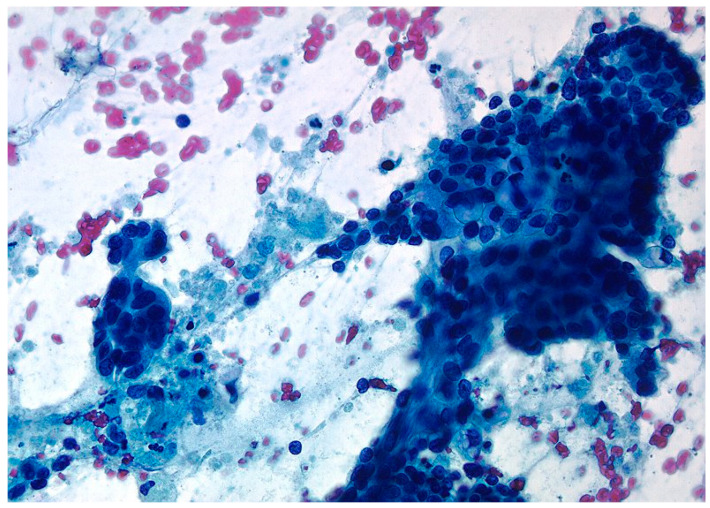
The tissue fragments show large cells with anisonucleosis, hyperchromasia, irregular nuclear membranes, and nuclear overlap, indicating high-grade ductal adenocarcinoma (Papanicolau 40×).

**Figure 3:**
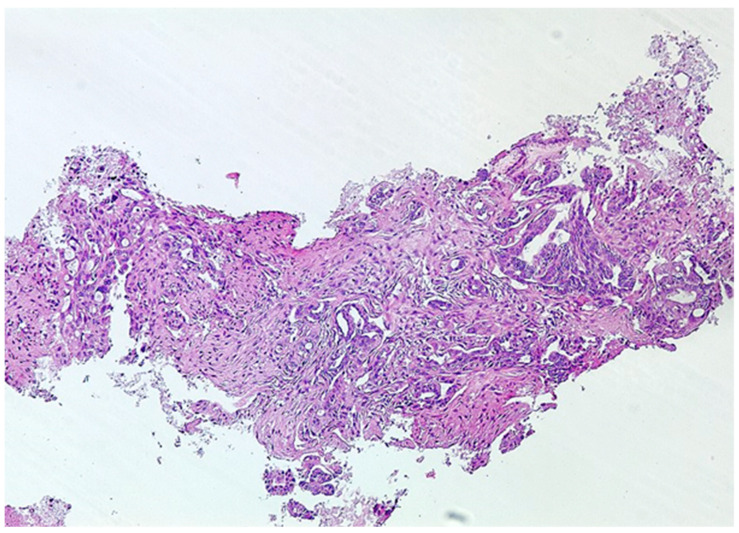
Ductal adenocarcinoma. Tissue fragments of neoplastic glandular cells showing irregular nuclei and mucinous cytoplasm with desmoplastic stroma (HE 20×).

The material obtained from EUS-guided tissue acquisition was processed as follows. If rapid on-site evaluation was available, cytological needles were used. Two or three smears were made from each needle pass, and one or two slides were air-dried and stained with Diff-Quick technique for rapid on-site evaluation. The remaining smears were fixed in 95% ethanol for later interpretation using Papanicolaou staining (Figure 2). The material remaining after puncture with cytology needles and the tissue samples obtained with core needles were placed in a preservative solution (Cytolit^®^ or Thinprep; Hologic^®^, Marlborough, MA, USA), in which the needle can also be rinsed. Sample management in the pathology department is based on filtration techniques to produce monolayer slides. This method produces slides in which the cells are evenly distributed over the surface. The same material is processed as a cell block and embedded in paraffin to produce tissue sections of 3 to 4 µm, which are stained with hematoxylin and eosin for morphological evaluation or subsequent immunohistochemistry or molecular studies (Figure 3).

### 2.3. Endpoints and Definitions

The primary endpoint was the diagnostic yield of EUS-guided tissue acquisition for the characterization of focal liver lesions. Secondary endpoints were the rate of adverse events and the evaluation of factors associated with the diagnostic yield of the technique.

Diagnostic yield was defined as the percentage of correct diagnoses obtained after EUS-guided tissue acquisition. For this purpose, the diagnoses made by the pathologist after processing and analyzing the specimen and the final diagnosis were recorded. For patients who underwent surgery, the final diagnosis was based on the histological evaluation of the surgical specimens. For patients who did not undergo surgery, the final diagnosis was based on the global clinical, laboratory, and imaging assessment using percutaneous US, multidetector computed tomography and/or magnetic resonance imaging, together with the cytohistological assessment, with a minimum follow-up of 6 months. 

A diagnosis of EUS-guided tissue acquisition is considered correct if the cytohistological specimen contains malignant or benign cells that are consistent with the final diagnosis and patient follow-up, irrespective of whether a specific diagnosis could be made. A puncture was deemed non-diagnostic if there was insufficient material for processing if an indeterminate diagnosis was reported, or if the cytohistological description did not match the final diagnosis and patient follow-up.

Adverse events were defined and graded according to the American Society of Gastrointestinal Endoscopy (ASGE) lexicon [12]. Events recorded in the electronic health record during the 6 months after the procedure were reviewed. The electronic health record is accessible to all doctors within the regional health system. It records all health events that occur to a patient.

### 2.4. Variables

Demographics and variables related to the characteristics of the focal liver lesions such as size and location were recorded. Variables related to the procedure such as indication for EUS-guided tissue acquisition, needle type (cytological or core needle) and size of the needle (G), number of needle passes, rapid on-site evaluation, and punctures from additional sites other than the liver during the EUS procedure were also collected.

### 2.5. Statistical Analysis

Data are shown as medians with interquartile range (IQR) for quantitative variables. Frequencies and percentages were calculated for categorical variables. The association between categorical variables was tested using Chi square test or Fisher’s exact test. Univariate and multivariate logistic regression was used to identify the factors significantly and independently associated with the diagnostic yield of EUS-guided tissue acquisition of focal liver lesions, and the results are expressed as OR and 95% CI. *p*-value < 0.05 was considered statistically significant. Data analysis was performed using SPSS Statistics 20. 

### 2.6. Ethical Aspects

The study was approved by the Clinical Research Ethics Committee of the Galician Ministry of Health (Comité Ético de Investigación Clínica de Galicia, Consellería de Sanidad, www.ceic.sergas.es, on 18 January 2023), with the approval number 2023/440. All patients provided written informed consent to the study. The study protocol conforms to the ethical guidelines of the 1975 Declaration of Helsinki. The study was conducted following the Declaration of Helsinki and its amendments, and Good Clinical Practice guidelines.

## 3. Results

The study included a total of 114 cases, of which 64 were male (56.1%) with a mean age of 68.0 ± 11.3 years. The clinical characteristics of the patients and procedural data are shown in Table 1. Core (FNB) needles were used in most of the cases, *n* = 72 (63.2%), whereas in 42 (36.8%) cases, cytological (FNA) needles were used. A rapid on-site evaluation was available in 28 cases (24.6%).

A sampling of a focal liver lesion previously detected using imaging was the indication for EUS-guided tissue acquisition in 56 cases (49.1%), 48 of them (85.7%) due to the location in the left liver lobe and 8 (14.2%) after the failure of a US-guided percutaneous biopsy. In the remaining 58 patients (50.9%), the focal liver lesion was first detected during EUS. The indication of EUS in these cases was the diagnosis and staging of pancreatic cancer (*n* = 29, 25.5%), staging of gastric cancer (*n* = 8, 7.0%), obstructive jaundice or persistent cholestasis (*n* = 7, 6.1%), and others (*n* = 14, 12.3%).

### 3.1. Diagnostic Yield

The final diagnosis of the focal liver lesions was the metastasis of pancreatic cancer (*n* = 39, 34.2%), metastasis of gastrointestinal cancer (*n* = 18, 15.7%), metastasis of lung cancer (*n* = 12, 10.5%), or metastasis of a pancreatic neuroendocrine tumour (*n* = 8, 7.0%), cholangiocarcinoma (*n* = 12, 10.5%), and a primary hepatic tumour (*n* = 4, 3.5%%). Other less frequent diagnoses were reported in the remaining 21 patients (18.4%). A correct cytohistological diagnosis could be made after EUS-guided tissue acquisition in 100 of these 114 cases (87.7%).

The additional punctures of lesions other than focal liver lesions were performed during the same EUS procedure in 40 cases (35.1%). This included 8 of the 14 cases in which the EUS-guided tissue acquisition of the focal liver lesion was not diagnostic and allowed the final diagnosis to be made in 7 of these cases (Table 2). The final diagnostic yield of EUS-guided tissue acquisition was therefore 93.9% (107 correct diagnoses in 114 patients).

No early or late adverse events according to the ASGE lexicon were reported. Self-limited bleeding at the puncture site was observed in one patient but did not require intervention. 

### 3.2. Factors Associated with the Diagnostic Yield of EUS-Guided Tissue Sampling

No significant differences were detected in the diagnostic yield comparing groups on different the variables evaluated (needle type, needle size, localization of focal liver lesion, rapid on-site evaluation, number of passes and lesion size) (Table 3). 

When analyzing the characteristics of focal liver lesions, in terms of location and size, no differences were detected (*p* = 0.841 and *p* = 0.921) (Table 3).

The needle type and size, number of needle passes, and rapid on-site evaluation were not significantly associated with the diagnostic yield of EUS-guided tissue acquisition in the univariate and multivariate analysis (Table 4).

## 4. Discussion

The present study demonstrates that EUS-guided tissue acquisition can safely obtain tissue samples adequate for the cytohistological evaluation of focal liver lesions. EUS-guided tissue acquisition could be considered as a first option access for focal liver lesions that are first detected using EUS, or for focal liver lesions that are more accessible using EUS than by any type of percutaneous techniques.

Endoscopic ultrasonography has become an essential tool for diagnosing and treating various gastrointestinal and extra-intestinal diseases. This includes pancreatic and biliary diseases, intraluminal tumors, and mediastinal diseases. In this period, the number of indications for EUS has increased over time as endosonographers have gained experience and new and better devices, such as the new core needles, have become available. Due to the proximity of the EUS transducer to the liver and the low rate of adverse events, EUS is a highly suitable modality for the diagnosis and staging of primary malignant liver tumors and metastatic liver disease [13]. Some studies have shown the role of EUS-guided advanced imaging in managing liver disease, including parenchymal diffuse disease and evaluating solid liver lesions [14]. EUS-guided elastography can differentiate between benign and malignant focal liver lesions, but it cannot provide a specific diagnosis. The same is true for contrast-enhanced harmonic EUS, which behaves similarly to percutaneous US with contrast. These methods provide valuable information for optimizing the detection of very small lesions. With all this information obtained, the differential diagnosis of focal liver lesions is mainly based on clinical history, risk factors, laboratory tests and imaging studies, including computed tomography, positron emission tomography–computed tomography and magnetic resonance imaging, especially for lesions > 2 cm, but also EUS. The role of EUS-guided tissue acquisition in the diagnosis of focal liver lesions is currently not well standardized [15].

Several published studies have evaluated the role of EUS-guided tissue acquisition in the diagnosis of focal liver lesions [7,16,17,18,19,20,21,22], with reported diagnostic yields ranging from 75% to 100%. Although these studies report EUS-guided tissue acquisition as an accurate and safe technique that could be used as a first-line option for the cytohistological characterization of focal liver lesions, most of them are retrospective series and only two of them used the new core needles [17,22]. The largest retrospective cohort of patients included 167 cases in a multicenter approach and was published more than two decades ago, before the technological development of EUS imaging and the development of the newer EUS needles [19].

The present study shows the diagnostic yield of EUS-guided tissue acquisition for focal liver lesions in clinical practice using the latest EUS equipment and needles. The results of our study are consistent with those previously reported and show that EUS-guided tissue acquisition allows the collection of tissue samples suitable for the cytohistological diagnosis of focal liver lesions in 87.7% of cases. In addition, the ability of EUS to detect and sample other associated lesions increases the diagnostic yield of EUS-guided tissue acquisition by up to 93.9%. The EUS-guided tissue acquisition of focal liver lesions is a very safe technique, and no adverse events were reported in this study.

The quality of tissue samples obtained after EUS-guided tissue acquisition has improved significantly with the development and use of core needles, using either the fork tip or Franseen type. These needles provide samples that are adequate for histological, immunohistochemical, and molecular evaluation. Core needles have been shown to be superior to standard cytology needles in the evaluation of solid pancreatic lesions [23] and parenchymal liver disease [24]. Furthermore, a recent study has shown that core needles yield more adequate samples than FNA needles for the evaluation of focal liver lesions [25]. However, the present study has shown a similar adequacy of samples obtained via core and cytology needles for the cytohistological diagnosis of focal liver lesions.

Rapid on-site evaluation is another factor that should be considered to improve the diagnostic yield of EUS-guided tissue acquisition, as previously demonstrated in solid pancreatic lesions [26]. Recent studies have shown that the diagnostic yield with core needles is like that of cytological needles with rapid on-site evaluation [27]. The present study confirms the safety and this similar diagnostic yield of cytological needles with rapid on-site evaluation and core needles in the context of focal liver lesions. In agreement with the present and other recent studies [28], our current policy for EUS-guided tissue acquisition of focal liver lesions is to use core needles as the first option, while reserving the use of cytological needles with rapid on-site evaluation as a rescue procedure. The diagnostic yield of EUS-guided tissue acquisition in focal liver lesions was not influenced by needle size. The most commonly used needles for this purpose are 22 gauge and 25 gauge, which have similar abilities to provide adequate samples for cytohistological evaluation. For studying parenchymal liver diseases via EUS liver biopsy, 19-gauge needles are primarily used [28,29]. This is because the quality of the sample is crucial for accurate assessment [8], and the puncture margin is typically wider than for a focal liver lesion.

Few published studies exist comparing the diagnostic accuracy of EUS-guided tissue acquisition with traditional percutaneous biopsy in focal liver lesions. In the recent study by Takano et al. [30], involving 106 patients (47 in the percutaneous group and 59 in the EUS group), EUS-guided tissue acquisition demonstrated similar accuracy and yielded samples of comparable quality to percutaneous biopsy, but with a lower rate of adverse events. Additionally, the EUS method may offer benefits such as shorter patient recovery time, lower levels of anxiety, and the ability to perform a gastrointestinal endoscopic and biliopancreatic endosonographic examination simultaneously with the biopsy (8).

Genomic profiling through next-generation sequencing is being increasingly utilized to deliver precision medicine for a range of advanced cancers. Concerns exist regarding the adequacy of samples acquired via EUS for molecular marker analysis. However, recent research supports the feasibility of this approach. Choi et al. performed EUS-guided tissue acquisition using core needles for solid liver cancers in the left lobe [31]. In this study, 12 patients had primary liver cancer, including four cases of hepatocellular carcinoma and seven of intrahepatic cholangiocarcinoma, while 16 had metastatic liver cancer. Genomic profiling analysis was conducted on 16 of these cases (57%), revealing KRAS mutations. Thus, although few actionable mutations lead to treatment for hepatocellular carcinoma, the genetic analysis of EUS-guided FNA/FNB specimens proves beneficial for metastatic liver cancer, especially for metastatic pancreatic or biliary tract cancer, as it identifies actionable mutations that can guide genome-matched therapy [32] 

Our study has important strengths. It is the largest series of patients undergoing EUS-guided tissue acquisition for the evaluation of focal liver lesions, representing current clinical practice in terms of current EUS imaging and needles. Despite the retrospective nature of the analysis, the data were obtained from a prospectively collected registry with very clear and strict outcome definitions. The single-center design, the fact that the study was carried out in a tertiary referral center for liver transplantation and that EUS-guided tissue acquisition was performed by highly experienced endosonographers must be considered as limitations of our study. Therefore, the results reported here may not be representative of clinical practice in smaller centers with less experience in advanced EUS. In this setting, prospective and multicenter studies will be desirable to establish the exact role of EUS-guided tissue acquisition in the management of patients with focal liver lesions, either already known and detected before the procedure in an imaging method or detected during the EUS examination.

## 5. Conclusions

EUS-guided tissue acquisition provides tissue samples that are highly suitable for the accurate cytohistological evaluation of focal liver lesions. EUS-guided tissue acquisition of focal liver lesions is a very safe technique that could be considered as an alternative to the percutaneous approach as the first option for focal liver lesions sampling. Neither needle type and size nor rapid on-site evaluation seems to affect the diagnostic yield of EUS-guided tissue acquisition in this setting.

## Figures and Tables

**Table 1:** Clinical characteristics of focal liver lesions and EUS-guided tissue acquisition procedure.

Total of FLL	114
Location of FLL, *n* (%)	
LHL	98 (85.9%)
RHL	16 (14.1%)
Size of FLL, median (Range)	20 mm (14.8–33.5)
Use of core needles (FNB), *n* (%)	72 (63.2%)
Franseen-type	11 (15.3%)
Fork-tip	26 (36.1%)
Reverse bevel	30 (41.7%)
Missing data	5 (6.9%)
Use of cytological needles (FNA), *n* (%)	42 (36.8%)
Number of needle passes, median (IQR)	1 (1–2)
ROSE, *n* (%)	28 (24.6%)

FNA = fine needle aspiration; FNB = fine needle biopsy; IQR = interquartile range; FLL = focal liver lesions; ROSE = rapid on-site evaluation.

**Table 2:** Extrahepatic EUS-guided tissue acquisition was performed during the EUS procedure in which the focal liver lesion was punctured. Cytohistological and final diagnosis are shown.

Extrahepatic Lesions Biopsy	Cytohistological Diagnosis	Final Diagnosis
Pancreatic mass (*n* = 4)	Adenocarcinoma (*n* = 3)	Metastasis of pancreatic ductal adenocarcinoma (*n* = 3)
	NET (*n* = 1)	Metastatis of pancreatic NET (*n* = 1)
Liver hilum lymph node	Carcinoma	Intrahepatic cholangiocarcinoma
Celiac axis lymph node	Gastrointestinal adenocarcinoma	Metastatis of colon cancer
Mediastinal lymph node (*n* = 2)	Biliopancreatic adenocarcinoma (*n* = 1)	Metastatic cholangiocarcinoma (*n* = 1)
	Sample insufficient for diagnosis (*n* = 1)	Metastasis of lung cancer (*n* = 1)

NET = neuroendocrine tumor.

**Table 3:** Variable factors tested for potential impact on diagnostic yield of EUS-guided tissue acquisition in solid liver lesions.

EUS-Guided Tissue Acquisition	Diagnostic Yield	*p*-Value	
Core needles (FNB)	25 G (25/28–89%)	*p* = 0.980 ^a^	*p* = 0.786 ^b^
	22 G (33/36–91%)		
	19–20 G (8/8–100%)		
Cytological needles (FNA)	25 G (17/22–77%)	*p* = 0.972 ^a^	
	22 G (16/19–84%)		
	19–20 G (1/1–100%)		
Focal liver lesion location			
Left liver lobe	85/98 (87%)	*p* = 0.841	
Right liver lobe	15/16 (94%)		
Rapid on-site evaluation			
Yes	27/28 (96%)	*p* = 0.684	
No	73/86 (85%)		
Number of passes			
1	58/66 (88%)	*p* = 0.992	
2	25/30 (83%)		
3	6/6 (100%)		
4	1/1 (100%)		
Focal liver lesion size			
≤20 mm	14/16 (88%)	*p* = 0.961	
>20 mm	9/10 (90%)		

EUS-TA = endoscopic ultrasound-guided tissue acquisition. FNA = fine needle aspiration; FNB = fine needle biopsy; G = gauge; a: comparison among different needle sizes; b: comparison between core and cytology needles.

**Table 4:** Factors associated with the diagnostic yield of EUS-guided tissue acquisition of focal liver lesions.

Diagnostic Yield	Univariate Analysis		Multivariate Analysis	
	OR (95IC)	*p*-Value	OR (95IC)	*p*-Value
Core needle	0.45 (0.09–2.15)	0.317	0.56 (0.03–9.66)	0.692
Number of passes	2.25 (0.65–7.84)	0.203	2.29 (0.65–8.09)	0.200
ROSE	4.57 (0.57–36.65)	0.153	2.67 (0.16–45.62)	0.497

OR: odds ratio; ROSE: rapid on-site evaluation.

## Data Availability

Data are unavailable due to privacy and ethical restrictions.
